# Systemic Inflammation and Metabolic Changes After Cardiac Surgery and Postoperative Delirium Risk

**DOI:** 10.3390/jcm14134600

**Published:** 2025-06-29

**Authors:** Kwame Wiredu, Jason Qu, Isabella Turco, Tina B. McKay, Oluwaseun Akeju

**Affiliations:** Mass General Brigham Department of Anesthesiology, Massachusetts General Hospital, 55 Fruit Street, Boston, MA 02114, USA

**Keywords:** cardiac surgery, postoperative delirium, metabolism, inflammation, proteomics

## Abstract

**Introduction:** Postoperative delirium (POD) remains a major complication in geriatric surgical care, with poorly understood molecular mechanisms. Emerging evidence links cardiac surgery to elevated markers of neurologic injury, even in cognitively intact individuals. While neuroinflammation is the prevailing model, a more detailed characterization of the systemic inflammatory and metabolic response to surgery may offer deeper insights into POD pathogenesis. **Methods:** We used the 7K SomaLogic proteomic platform to analyze preoperative and postoperative day-one serum samples from 78 patients undergoing cardiac surgery with cardiopulmonary bypass. We compared proteomic profiles within individuals (pre- vs. post-surgery) and between those who developed POD and those who did not. Functional analyses were performed to identify relevant biological pathways. A composite metabo-inflammatory score (MIF) was derived to quantify systemic derangement. We modeled the association between POD and age, sex, baseline cognition, and MIF score. **Results:** Cardiac surgery with CPB was associated with marked inflammatory responses across all subjects, including increased IL-6, CRP, and serum amyloid A. Compared to controls, POD cases showed greater metabo-inflammatory shifts from baseline (average logFC = 2.56, *p* < 0.001). Lower baseline cognitive scores (*OR* = 0.74, *p* = 0.019) and higher MIF scores (*OR* = 1.03, *p* = 0.013) were independently associated with increased POD risk. **Conclusions:** Cardiac surgery with CPB elicits a significant metabo-inflammatory response in all patients. However, those who develop POD exhibit disproportionately greater dysregulation.

## 1. Introduction

Postoperative delirium (POD) is a common and serious complication in older adults undergoing cardiac surgery with cardiopulmonary bypass (CPB). Characterized by acute disturbances in attention, cognition, and consciousness, POD is associated with increased morbidity, longer hospital stays, higher costs of care, and long-term cognitive decline [1,2,3,4]. Although reported incidence rates vary—likely due to differences in study design, including assessment timing and measurement tools—recent studies suggest that POD remains a prevalent complication in the cardiac surgical population, with rates typically reported between 10% and 30% [5,6,7,8]. Compared to other surgical populations, patients undergoing CPB appear particularly vulnerable, likely due to the added physiologic stress and inflammatory burden introduced by the extracorporeal circulation [9,10,11,12].

While inflammation is widely associated with POD [13,14,15], the broader biological response to surgery is increasingly appreciated [16,17]. Cardiac surgery leads to elevations in markers of neuronal injury and degeneration, including neurofilament light chain (NfL), glial fibrillary acidic protein, ubiquitin C-terminal hydrolase L1, TAR DNA-binding protein 43, and tau [18,19]. These increases reflect the activation of pathways involved in axonal injury, glial reactivity, protein degradation, RNA processing, and blood–brain barrier disruption. In parallel, metabolic disturbances such as oxidative stress and impaired glucose utilization have been implicated in postoperative delirium [20,21,22,23]. Notably, patients who develop POD often show elevated baseline levels of NfL and tau, suggesting a preexisting vulnerability that may interact with perioperative stress to precipitate delirium [18,19]. While neuroinflammation remains the prevailing hypothesis, a more integrated understanding of systemic inflammatory and metabolic responses to cardiac surgery may provide deeper insights into POD pathogenesis and individual susceptibility.

In this nested case–control study within a prospective cardiac surgery cohort, we used a high-throughput proteomic platform capable of quantifying the broadest range of proteins currently available to characterize metabo-inflammatory changes associated with cardiac surgery and postoperative delirium. By linking systemic biological signatures with clinical outcomes, we aimed to clarify how inflammation, metabolic dysregulation, and baseline vulnerability interact to influence POD risk.

## 2. Methods

### 2.1. Study Design and Patient Selection

**Figure 1:**
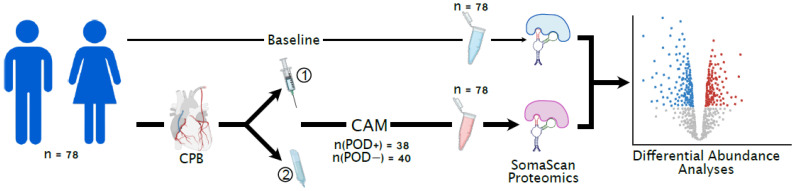
**Study design, data acquisition and analyses.** An age- and sex-matched cohort of 78 patients was selected from the parent MINDDS trial of 364 non-mechanically ventilated patients who underwent non-emergent cardiac surgery with cardiopulmonary bypass. The participants were randomized to receive either dexmedetomidine or placebo and were followed subsequently with twice-daily assessment for delirium using the confusion assessment method. Sera for each participant in this nested study was comprehensively profiled for proteomic signatures using the high-throughput 7K SOMAScan proteomic platform. Comparative proteomic analyses between delirium cases and non-delirium controls were performed for differentially abundant proteins, among other analyses. **CAM**: confusion assessment method; **CPB**: cardiopulmonary bypass; **POD+**: postoperative delirium case; **POD−**: non-POD control.

This is a nested case–control study of 78 subjects (Figure 1), selected from an original cohort of 394 participants from the parent randomized, placebo-controlled superiority trial [6,24]. A post hoc analysis (Supplementary Figure S1A), indicates 80% power at significance level, α = 0.05 to detect Cohen’s *d* ≥ 0.65 SD in biomarkers commonly reported delirium biomarkers such as IL-6.

The primary objective of the parent study was to determine the incidence of postoperative delirium after a sleep-inducing dose of dexmedetomidine in a population of patients, ≥60 years, who underwent non-emergent cardiac surgery with cardiopulmonary bypass. Patients were excluded from this trial if they had a documented allergy to dexmedetomidine, had liver or renal failure requiring dialysis, had cardiac surgery in the preceding one year, or did not speak English language. This study was approved by the Partners Healthcare Institutional Review Board, and registered with ClinicalTrials.gov (https://clinicaltrials.gov/ct2/show/NCT02856594; principal investigator: Oluwaseun Johnson-Akeju; registration date: 5 August 2016). Details of the study protocol are reported previously [24].

Cases of postoperative delirium (POD) were identified with twice-daily [25] use of the Confusion Assessment Method (CAM), a proven delirium screening tool with high sensitivity and specificity, (91–97% and 85–94%, respectively), and good interrater reliability (κ=0.92) [26]. Cases were matched to controls by age and biological sex, at an approximate ratio of 1:1. To reduce selection bias, the controls were selected irrespective of their treatment assignment from the parent MINDDS trial, such that the distribution of dexmedetomidine exposure among controls in this nested cohort (50% placebo, 50% dexmedetomidine) reflects the distribution of exposure in the parent MINDDS trial.

Briefly, tMoCA is a modified version of in-person MoCA, optimized for remote assessment of cognition [27,28].

### 2.2. Sample Collection

For each subject, blood samples were collected at baseline and on postoperative day one into a plastic serum separator tube (BD Vacutainer tube, BD Life Sciences, Franklin Lakes, NJ, USA) and allowed to clot for 30 min. After centrifugation at 383× *g* at 22 °C for 30 min, the supernatant was collected and stored at −80 °C prior to omics profiling.

### 2.3. Proteomic Profiling Using SOMAScan

Proteomic profiling was achieved using SOMAScan (SomaLogic, Boulder, CO, USA), a high-throughput, aptamer-based platform, capable of simultaneously measuring about 7000 human proteins [29,30]. The choice of an aptamer for a given protein is based on the binding affinity of the sequence of nucleic acids to the protein molecule. The aptamers are further modified to ensure limited chemical diversity, lower possibility of cross-reactivity and enhanced binding specificity [31]. The platform is sensitive (up to about 40 femtomoles median lower limit of detection) and has specificity that is superior to that of traditional affinity-based methods [32]. Further, the SOMAScan platform is complimentary to mass spectrometry, most especially for proteins in the low-abundance proteome [33].

To cover the entire dynamic range of the human proteome and reduce signal suppression by highly abundant proteins such as albumin, 70 µL of each of the 171 patient samples was analyzed in three dilutions bins. Protein quantification on the platform was based on the addition of a set of hybridization control sequences, calibrated to report measurements in relative fluorescence units (RFUs). Using a total of two 96-well plates, equal amounts of a pooled human sera were randomly added to each plate of samples to account for plate-to-plate variations. The acquired dataset was exported as a comma separated values (.csv) file for downstream statistical analyses.

### 2.4. Bioinformatics and Statistical Analyses

All analyses were performed in R language for statistical computing (v4.2.0) [34] at a significance level of alpha ≤ 0.05.

Baseline characteristics: Descriptive statistics on baseline characteristics of the study participants are reported as mean (±standard deviation) and count (percentage) for continuous and categorical variables, respectively. For Patient-Reported Outcome Measurement Information System (PROMIS) responses, the corresponding t-scores were categorized according to the domain and sub-domains appropriate for adults over 60 years [35,36]. The significance test for continuous variables was achieved using Student’s *t*-test (assuming unequal variance) and Chi-squared analyses for categorical variables.

Normalization and proteomic feature selection: Protein measurements from the three dilution bins (i.e., 20:1, 0.5:1 and 0.005:1) were first normalized to a common dilution ratio. This was followed by quantile normalization to align samples to a comparable distribution scale [37,38], based on the tested assumption that most proteins in a sample matrix remain relatively unchanged. Given that systematic plate-to-plate differences existed in protein quantification, batch effect was corrected for using the ComBat algorithm, which employs an empirical Bayes approach to remove technical variation (i.e., plate-to-plate differences in this case) by standardizing the means and variances between batches/plates based on batch covariate information [39,40].

Important protein features between pre- and post-CPB proteome, and between POD cases from non-cases, were identified using the Elastic Net algorithm [41]. Briefly, Elastic Net is a regularization and feature selection method that imposes a penalty on features based on their contribution in predicting the outcome variable in question. Elastic Net combines the advantages of Lasso and Ridge regression methods, performs well on high-dimensional data (i.e., data with large *p* but small *n* in an *n* × *p* matrix), is insensitive to highly abundant protein features such as albumin that are typically dominant in the matrix, and overcomes the problems of multicollinearity [42,43,44]. Optimal hyperparameters for each Elastic Net model were identified by grid optimization, after which they were used to build all final models. All final models were validated using leave-one-out cross-validation and model performances were assessed using the metrics of (1) misclassification error rate and (2) prediction accuracy on the hold-out set.

Differential abundance and logistic regression: Following feature selection, differential abundance analyses were performed on the subset of important proteomic features obtained from the Elastic Net modeling, herein referred to as the metabo-inflammatory profile of the study subjects. Here, a moderated *t*-test, that also relies on empirical Bayes, was employed. The average pre- and post-CPB log-fold change was computed for both cases and controls.

Lastly, logistic regression analysis was performed, regressing age, biological sex, baseline neurocognitive function (as measured by tMoCA) and the metabo-inflammatory profiles of subjects on POD outcome. In order to perform this regression analyses, differentially abundant postoperative protein biomarkers that reached statistical significance were used to compute a single, composite “pseudo-protein” value that represents the metabo-inflammatory profile (i.e., the MIF score) for each study subject. This composite biomarker score, constrained between −1 and 1 per protein, was achieved by assuming a linear relationship between the biomarkers and summing up their standardized protein values as follows:

$$\frac{x_{{ij}_{1}-\mu_{j_{1}}}}{\sigma_{j_{1}}}+\frac{x_{{ij}_{2}-\mu_{j_{2}}}}{\sigma_{j_{2}}}+\cdots +\frac{x_{{ij}_{n}-\mu_{j_{n}}}}{\sigma_{j_{n}}},$$

where *x_ij_*_1_ is the normalized abundance value of biomarker *j*_1_ for the study subject *x_i_*, *µ_j_*_1_ is the mean protein abundance values for biomarker *j*_1_, and *σ_j_*_1_ is the standard deviation of the protein values for biomarker *j*_1_.

Data visualization: Inherent data structures and clusters within data were visualized using the first 2 components from principal component analyses. Volcano plots were used to display differential abundance testing at varied fold-change cutoffs. Functional enrichment analyses were used for over-represented biological functions, subcellular components, and metabolic pathways. The average pre-/post-CPB log-fold changes were illustrated in bar charts for both cases and controls.

## 3. Results

### 3.1. Baseline Subject Characteristics

The clinical profile of the subjects in this nested case–control study (*n* = 78) is presented in Table 1. There were no significant differences between POD cases and controls with respect to age, sex, prevalence of diabetes mellitus, self-reported health metrics, intraoperative indices (such as duration of CPB) or postoperative events (such as readmission rates). We, however, found that POD cases had a significantly lower baseline neurocognitive function than non-POD controls (*µ*_tMoCA_: 17 versus 19, *p* = 0.002), and a higher ICU length of stay (average of 55 versus 34 h, *p* = 0.04).

### 3.2. Signatures of CPB Exposure

**Figure 2:**
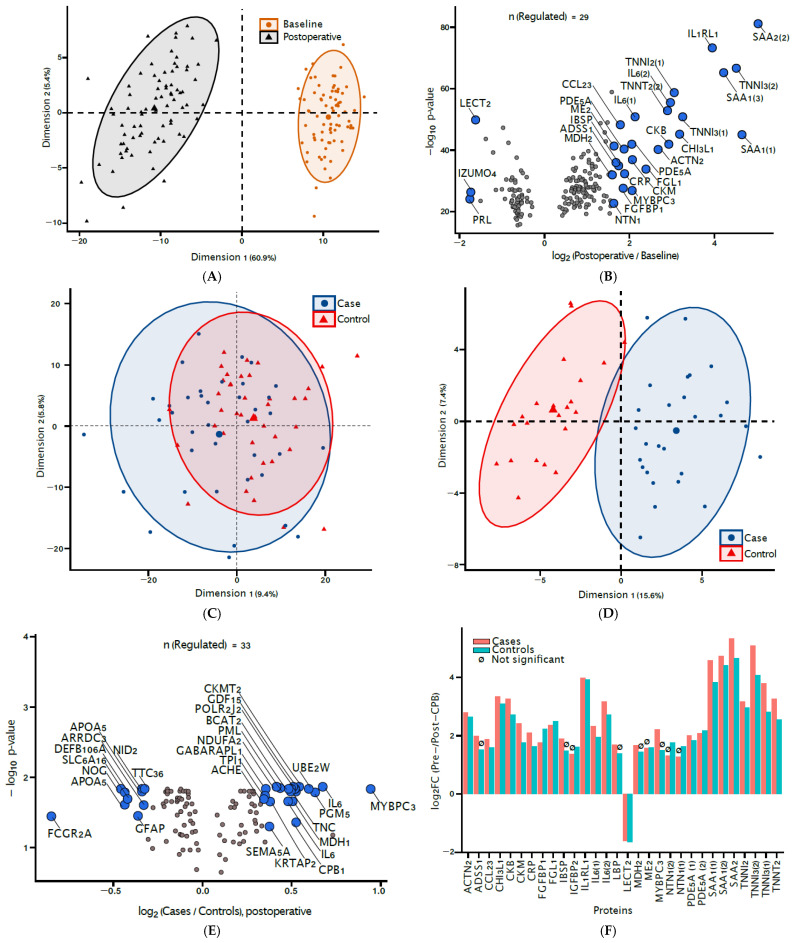
(**A**) Principal component analyses (PCA) of all baseline and postoperative day-one samples, based on 202 protein features identified by cross-validated penalized logistic regression (Elastic Net) modeling. (**B**) Volcano plot of *p*-value (−log_10_ scale) vs. fold-change (log_2_ scale) of the 29 differentially abundant proteins, showing a four-fold postoperative change in select proteins, notably SAA1, SAA2, IL1RL1 and TNNI3, relative to baseline levels. (**C**) PCA of all samples at baseline, annotated by case–control status. (**D**) PCA of all samples on postoperative day one, annotated by case–control status. (**E**) Volcano plot of the 33 differentially abundant proteins among POD cases relative to controls at postoperative day one, which demonstrates significant changes in many markers of acute inflammation in addition to enzymes involved in energy metabolism. (**F**) Bar plot depicting the fold changes on postoperative day one relative to baseline for the 30 most differentially abundant proteins between POD cases and controls. Only the top 30 proteins were selected to facilitate clear visualization. ***n*(Regulated)**: the number of proteins that reach statistical significance and the fold-change cut off for a given analysis. A4GNT: Alpha-1,4-N-acetylglucosaminyltransferase; ACHE: Acetylcholinesterase; ACTN2: Alpha-actinin-2; ALOX15B: Polyunsaturated fatty acid lipoxygenase ALOX15B; APOA5: Apolipoprotein A-V; ARRDC3: Arrestin domain-containing protein 3; BCAT2: Branched-chain-amino-acid aminotransferase, mitochondrial; CCL23: C-C motif chemokine 23; CHI3L1: Chitinase-3-like protein 1; CKB: Creatine kinase B-type; CKM: Creatine kinase M-type; CKMT2: Creatine kinase S-type, mitochondrial; CPB1: Carboxypeptidase B; CRP: C-reactive protein; DEFB106A: Beta-defensin 106; FCGR2A: Immunoglobulin gamma Fc region receptor II-a; FGFBP1: Fibroblast growth factor-binding protein 1; FGL1: Fibrinogen-like protein 1; GABARAPL1: Gamma-aminobutyric acid receptor-associated protein-like 1; GDF15: Growth/differentiation factor 15; GFAP: Glial fibrillary acidic protein; IGFBP2: Insulin-like growth factor-binding protein 2; IL1RL1: Interleukin-1 receptor-like 1; IL6: Interleukin-6; IZUMO4: Izumo sperm-egg fusion protein 4; LECT2: Leukocyte cell-derived chemotaxin-2; MDH1: Malate dehydrogenase, cytoplasmic; MYBPC3: Myosin-binding protein C, cardiac-type; NDUFA2: NADH dehydrogenase [ubiquinone] 1 alpha subcomplex subunit 2; NID2: Nidogen-2; NOG: Noggin; NQO2: Ribosyldihydronicotinamide dehydrogenase [quinone]; NTN1: Netrin-1; PBX1: Pre-B-cell leukemia transcription factor 1; PDE5A: cGMP-specific 3′,5′-cyclic phosphodiesterase; PGM5: Phosphoglucomutase-like protein 5; PML: Protein PML; POLR2J2: DNA-directed RNA polymerase II subunit RPB11-b1; PRL: Prolactin; SAA1: Serum amyloid A-1 protein; SAA2: Serum amyloid A-2 protein; SEMA5A: Semaphorin-5A; SFRP5: Secreted frizzled-related protein 5; SLC6A16: Orphan sodium- and chloride-dependent neurotransmitter transporter NTT5; TNC: Tenascin; TNNI2: Troponin I, fast skeletal muscle; TNNI3: Troponin I, cardiac muscle; TNNT2: Troponin T, cardiac muscle; TPI1: Triosephosphate isomerase; TTC36: Tetratricopeptide repeat protein 36; UBE2W: Ubiquitin-conjugating enzyme E2 W.

Feature selection for signatures of CPB exposure revealed 202 proteins. A principal component analysis (PCA, Figure 2A) confirmed the discriminating power of the 202 proteins with non-zero contribution in distinguishing baseline and post-CPB proteomic profiles. The most differentially abundant signatures of CPB exposure were markers of acute inflammation such as interleukin 6 (IL-6), c-reactive protein (CRP) and various isoforms of serum amyloid A (SAA) (Figure 2B). Markers of cardiac tissue damage, including troponin I (TNNI3), troponin T (TNNT2), and the muscle type of creatine kinase (CKM), were also observed.

### 3.3. Biomarkers of Delirium

We compared the sera of POD cases and non-POD controls at baseline and at postoperative day one (Figure 2C,D). At baseline, the subjects were indistinguishable by their proteomic profiles alone (Figure 2C). However, postoperatively, the POD cases exhibited modest but distinct proteomic profiles from their non-POD counterparts (Figure 2D). At a fold-change of at least ±log_2_ (1.25) between the cases and the controls, we identified 33 differentially abundant proteins, including cytoplasmic malate dehydrogenase (MDH1), carboxypeptidase B (CPB1), mitochondrial Creatine kinase S-type (CKMT2), and other enzymes involved in energy metabolism (Figure 2E).

Further, we compared whether the post-CPB changes described above were any different between the cases and the controls. Here, we observe that cases had a significantly different metabo-inflammatory response for an overlapping set of 30 protein biomarkers (Figure 2F). Of note, although the levels of IL-6, for example, were differentially abundant in all subjects postoperatively, a higher degree of change was detected among cases than in the controls for all of its isoforms.

**Figure 3:**
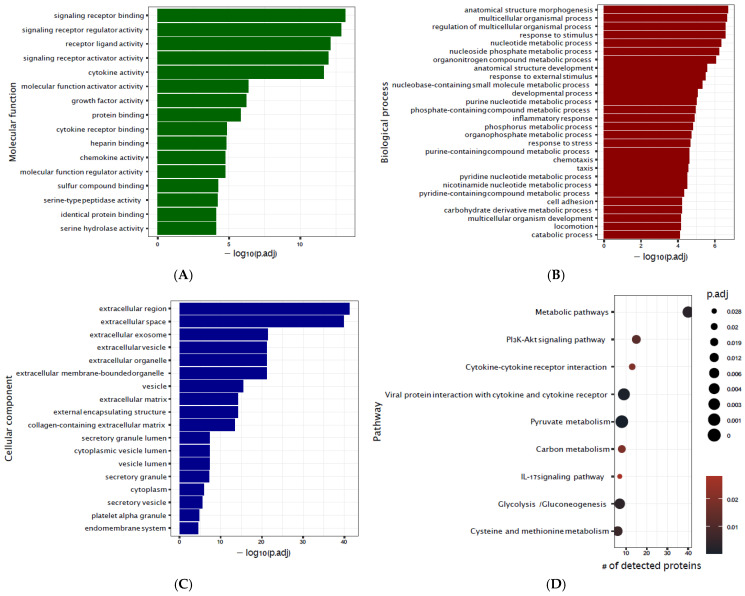
Functional analysis on protein signatures of CBP exposure and of delirium, for overrepresented (**A**) molecular functions, (**B**) biological processes, (**C**) subcellular components and (**D**) KEGG pathways.

Lastly, enrichment analyses for biological processes, molecular functions, subcellular components and overrepresented pathways revealed differentially abundant proteins that were largely extracellular, and involved in cell-to-cell signaling, inflammatory responses and metabolic pathways (Figure 3A–D).

### 3.4. Metabo-Inflammatory Model of Delirium

Based on the observations above, we hypothesized that a metabo-inflammatory model offers a more complete construct of delirium. To achieve this, we created a composite pseudo-protein profile for each subject, the value of which represents their respective postoperative metabo-inflammatory response (MIF score). An ROC analyses of the MIF scores alone showed an area under the ROC curve of 0.714 (Supplementary Figure S1B). Using unconditional logistic regression, we modeled metabo-inflammation (MIF) as a predictor of POD, adjusting for age, biological sex, baseline neurocognitive function and dexmedetomidine treatment effect (Table 2). The subjects were 26% less likely to develop POD for each unit increase in baseline tMoCA score, and subjects who received dexmedetomidine were 74% less likely to have exhibit delirium. Consistent with prior observations, however, subjects with higher postoperative day 1 metabo-inflammatory scores had higher odds of delirium than their low-scoring counterparts.

## 4. Discussion

In this study, we used high-throughput proteomics to characterize systemic biological responses to cardiac surgery with CPB. While markers of inflammation such as IL-6 and CRP were elevated across all patients and there was no difference in the prevalence of diabetes between POD cases and controls, only those who developed POD exhibited a distinct and exaggerated metabo-inflammatory profile on postoperative day one. These differences were not evident at baseline, suggesting that it is the magnitude of the postoperative dysregulation that differentiates POD cases. Importantly, these changes were most pronounced in patients with a lower baseline cognitive function, supporting the view that preexisting vulnerability amplifies the impact of surgical stress. Together, these findings reinforce our hypothesis that the interaction between inflammatory and metabolic pathways offers a more comprehensive framework for understanding the pathophysiology of POD.

The upregulation of acute inflammatory markers in our study, notably IL-6, CRP, and SAA following CPB exposure, is consistent with the neuroinflammation model of delirium [15,45,46]. For instance, Taylor et al. [46] demonstrated that the resolution of elevated IL-6 after surgery correlates with the return of normal cognitive function. However, our finding that this inflammatory response occurred in all patients, regardless of POD status, suggests that inflammation alone does not fully account for delirium risk. Instead, the distinctive metabolic profile of POD cases—reflected in the differential abundance of proteins involved in energy metabolism, such as cytoplasmic malate dehydrogenase, carboxypeptidase and mitochondrial Creatine kinase S-type—points to a broader pathophysiological process. This finding, together with others [21,22], supports the emerging concept of “metabo-inflammation,” characterized as a chronic, low-grade systemic inflammation driven by metabolic disturbances [22]. Although the baseline inflammatory levels did not differ between POD cases and controls in our cohort, this does not rule out the possibility of an elevated baseline inflammatory state in our cohort overall. Notably, the observation of postoperative metabolic dysregulation alongside a systemic inflammatory response on postoperative day one provides new insights into the evolving hypothesis of POD, suggesting that surgical insults in cognitively vulnerable individuals with a potentially heightened preoperative inflammatory state [15,47,48] may precipitate POD through concomitant metabolic dysregulation—a process consistent with the concept of “metabo-inflammation”.

To better capture the systemic biological disruption associated with POD, we developed the MIF score as a composite index reflecting the postoperative metabo-inflammatory response. The observed association between higher MIF scores and postoperative delirium suggests that it is the magnitude of the postoperative response, rather than its simple presence, that distinguishes patients at risk. Also, individuals with lower cognitive performance prior to surgery were more likely to develop POD, consistent with the concept that a reduced cognitive reserve limits resilience to perioperative stressors [49,50]. While our analyses suggest that the observed proteomic differences are independently associated with outcome, it is equally likely that these differences may simply reflect underlying neurocognitive vulnerabilities mediating the outcome of delirium. In fact, some but not all of the proteomic signatures identified in our study, such as MDH1 [4,5], CPB1 [6] and IL6 [7], have been reported elsewhere as markers of chronic cognitive impairment. Notwithstanding this possible confounding, the metabo-inflammatory dysregulation seen in our cohort was only apparent postoperatively, which makes it more likely to be a reflection of the impact of perioperative exposures on the vulnerable brain, rather than solely a chronic pathology. Together, these findings underscore the importance of incorporating both biological and cognitive dimensions into future models for POD risk stratification and prevention.

Our study has several limitations. First, the sample size may have limited our ability to detect subtle or heterogeneous proteomic differences. This secondary analysis aimed to elucidate broad metabo-inflammatory dysregulation patterns rather than nuanced biomarker-specific interactions. Nonetheless, a post hoc analysis (Supplementary Figure S1A) confirms 80% power (α = 0.05) to detect Cohen’s *d* ≥ 0.65 SD, aligning with observed effect magnitudes in key biomarkers (e.g., IL-6 log2FC = 3.1). Further, we applied rigorous statistical methods to strengthen the validity of our findings. Second, while we identified associations between postoperative metabo-inflammatory burden and POD, the observational design of this study limits causal inference. Third, we did not replicate previously reported baseline differences in tau and NfL between POD cases and controls. This may reflect limitations in the sensitivity of the SomaScan platform for detecting low-abundance proteins in serum. Lastly, while cognitive vulnerability and metabo-inflammatory burden independently predicted delirium, we did not test a statistical interaction between the two. Nonetheless, the conceptual relationship between cognitive vulnerability and heightened sensitivity to systemic stress remains compelling and warrants further investigation. Despite these limitations, our study provides novel insights into the systemic biological response to cardiac surgery and highlights promising avenues for improving risk stratification and understanding the pathophysiology of delirium.

In summary, our findings highlight that while systemic inflammation is a consistent consequence of cardiac surgery with CPB, it is the scale and character of the postoperative biological response, inclusive of metabolic processes, that distinguishes patients who develop delirium. By integrating high-throughput proteomics with clinical phenotyping, we identified a distinct postoperative signature associated with POD and suggest composite measures may aid in future risk stratification. These results support a more nuanced understanding of POD pathophysiology and provide a foundation for developing biologically informed strategies to better predict, prevent, and manage delirium in vulnerable surgical populations.

## Figures and Tables

**Table 1:** Baseline characteristics of study participants.

	Case (N = 38)	Non-Case (N = 40)	*p*-Value
Age (years)	72 (±6.2)	70 (±6.1)	0.174
Biological sex			
Female	18 (47%)	19 (48%)	1.000
Male	20 (53%)	21 (52%)	
BMI (Kg/m^2^)	28 (±4.8)	30 (±5.4)	0.186
Baseline neurocognition (MoCA scores)	17 (±3.3)	19 (±2.0)	0.002
Treatment			
Dexmedetomidine	9 (24%)	17 (42%)	0.128
Placebo	29 (76%)	23 (58%)	
PROMIS Physical health			
Poor	8 (21%)	2 (5%)	0.196
Fair	4 (11%)	9 (22%)	
Good	11 (29%)	10 (25%)	
Very good	10 (26%)	12 (30%)	
Excellent	5 (13%)	7 (18%)	
PROMIS Mental health			
Fair	4 (11%)	2 (5%)	0.236
Good	8 (21%)	3 (8%)	
Very good	15 (39%)	21 (52%)	
Excellent	11 (29%)	14 (35%)	
PROMIS Pain interference			
Moderate	5 (13%)	4 (10%)	0.809
Mild	8 (21%)	7 (18%)	
Normal	25 (66%)	29 (72%)	
PROMIS Applied cognition			
Severe	1 (3%)	1 (2%)	0.978
Moderate	6 (16%)	5 (12%)	
Mild	4 (11%)	4 (10%)	
Normal	27 (71%)	30 (75%)	
Duration of CPB (mins)	140 (±53)	130 (±47)	0.131
Cross-clamp time (mins)	100 (±42)	93 (±35)	0.228
Duration of surgery (hours)	6.3 (±1.5)	6.0 (±1.2)	0.390
Hospital length of stay (days)	8.2 (±5.5)	6.5 (±1.9)	0.073
Duration of Ventilation (hours)	10 (±19)	6.5 (±6.7)	0.282
ICU Length of Stay (hours)	55 (±56)	34 (±22)	0.040
Discharge location *			
Extended care ^†,^*	15 (39%)	8 (20%)	0.086
Home	22 (58%)	32 (80%)	
Hospital readmission *			
No	33 (87%)	37 (92%)	0.914
Yes	4 (11%)	3 (8%)	
ICU readmission			
No	35 (92%)	39 (98%)	0.571
Yes	3 (8%)	1 (2%)	

*: Discharge location and Hospital readmission were each missing for 1 participant. ^†^: extended care include traditional care units and rehabilitation facilities.

**Table 2:** Logistic regression analyses for POD outcome.

Variable	Beta	Std Error	Odds Ratio	*p* Value
Age (*in years*)	−0.025	0.04	0.97	0.600
Sex (*male*)	0.126	0.54	1.13	0.820
Baseline neurocognition (*tMoCA*)	−0.303	0.13	0.74	**0.019**
Composite biomarker profile	0.012	0.01	1.03	**0.013**
Treatment (Dexmedetomidine)	−1.358	0.62	0.26	**0.029**

tMoCA: telephonic Montreal Cognitive Assessment tool. Bold: Statistically significant.

## Data Availability

Full datasets as used in this study are available upon request.

## Supplementary Materials

The following supporting information can be downloaded at: https://www.mdpi.com/article/10.3390/jcm14134600/s1.
