# Supplementary figures and images for: Systemic Inflammation and Metabolic Changes After Cardiac Surgery and Postoperative Delirium Risk
Source: J Clin Med. 2025 Jun 29;14(13):4600. doi: 10.3390/jcm14134600 (PMC12251062; doi:10.3390/jcm14134600)

**A****Two-sample t test power calculation**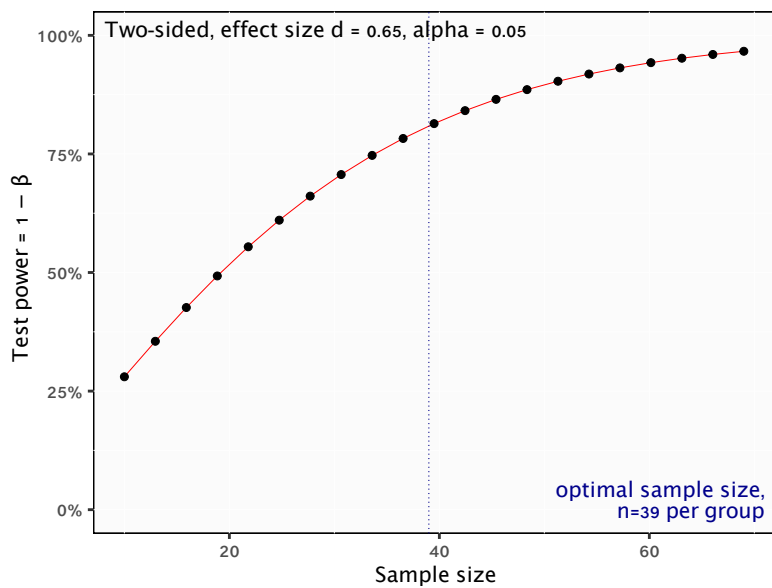**B****POD Prediction Based on MIF Score**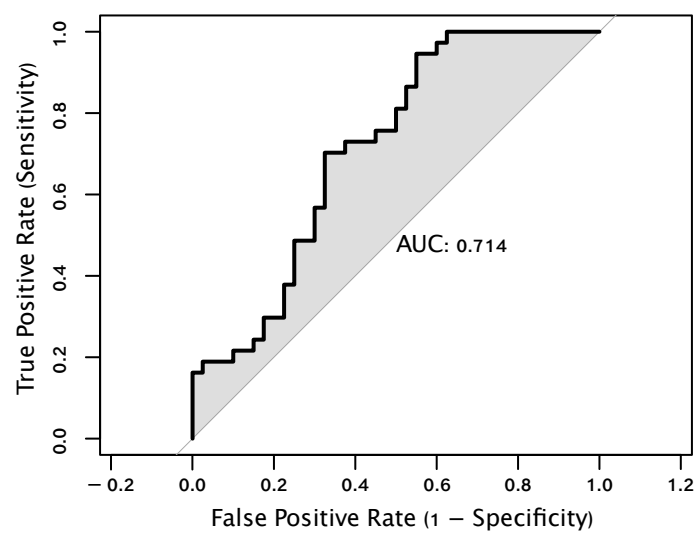**Supplemental Figure S1**

Supplement: Supplementary file 1 [file jcm-14-04600-s001.zip › jcm-3665696-supplementary.pdf]
